# RUSH to the Diagnosis: Identifying Occult Pathology in Hypotensive Patients

**DOI:** 10.5811/cpcem.20315

**Published:** 2024-09-14

**Authors:** Matt Berger, Jamal Hussain, Marco Anshien

**Affiliations:** Capital Health Emergency Medicine, Department of Emergency Medicine, Trenton, New Jersey

**Keywords:** point-of-care ultrasound, RUSH exam, cardiac tamponade

## Abstract

**Case Presentation:**

A 63-year-old female presented to our emergency department with altered mental status and hypotension. She was transferred from the outpatient interventional radiology suite after becoming unresponsive during the removal of an inferior vena cava filter. The patient arrived somnolent with no other history available. Her physical exam was unremarkable. We used point-of-care-ultrasound to perform a rapid ultrasound for shock and hypotension (RUSH) exam. A large pericardial effusion along with signs of cardiac tamponade were identified. The cardiothoracic surgery team was notified, and the patient was taken to the operating room where pericardial blood and a large hematoma were evacuated. She recovered uneventfully and was discharged one week later.

**Discussion:**

The above case describes a very unstable patient whose diagnosis was obtained using the RUSH exam. History and physical did not point to a clear etiology. Options were very limited. She was too unstable to go for computed tomography, and other tests such as electrocardiogram, chest radiograph, and lab work would have been non-diagnostic. It was only after the cardiac view of the RUSH exam was obtained that a pericardial effusion and developing tamponade were identified, facilitating timely management. The RUSH exam, like the extended focused assessment with sonography for trauma, is used to help determine pathologies that need immediate intervention. Incorporation in the evaluation of critically ill patients reduces the time to diagnosis. Our case is a unique example of how point-of-care ultrasound can be used to urgently identify a life-threatening pathology.

## CASE PRESENTATION

A 63-year-old female with multiple comorbidities presented as a rapid response from the outpatient interventional radiology suite. During removal of an inferior vena cava (IVC) filter, she became hypotensive and unresponsive. She received reversal agents for her sedation, glucose, and a bolus of fluid with mild improvement. Upon arrival to the emergency department (ED), she remained severely hypotensive, and aggressive fluid resuscitation was initiated. Initial vital signs on arrival to the ED included blood pressure 77/59 millimeters of mercury, heart rate 76 beats per minute, respiratory rate 11 breaths per minute, pulse oximetry 100% on 2 liters nasal canula, and temperature 36.7° Celsius.

The patient was somnolent and confused but had normal work of breathing, and her abdomen was soft and nontender. She had weak peripheral pulses. Given her critical state, further physical exam was postponed in favor of a rapid point-of-care ultrasound. We performed a point-of-care rapid ultrasound for shock and hypotension (RUSH) exam (Video), which identified a large pericardial effusion with signs of cardiac tamponade and possible proximal IVC injury or thrombus. The cardiothoracic surgery team was immediately notified, and the patient was taken emergently to the operating room where pericardial blood and a large hematoma were evacuated with immediate return of normal cardiac function.

The operative report confirmed an area of bruising to the right atrial appendage that was discovered after hematoma evacuation. This was identified as the site of right atrial perforation from the IVC filter removal wire that had caused the acute cardiac tamponade to develop. The patient was transferred to the intensive care unit. Postoperative computed tomography did not identify further injuries other than those mentioned in the operative report but did identify a small amount of hemoperitoneum, which potentially supported the possible IVC injury identified on RUSH exam. She recovered and was discharged to a rehabilitation facility on postoperative day seven.

## DISCUSSION

The above case describes how a very unstable patient was diagnosed rapidly with point-of-care ultrasound using the RUSH exam. On arrival, the cause of the patient’s hypotension was unknown. History did not provide any further information, and physical exam was remarkable for only hypotension; muffled heart sounds or jugular veinous distention were not present. It was only with the cardiac view of the RUSH exam that a pericardial effusion and developing tamponade were identified. Options in this case were very limited. She was too unstable to send to radiology, and labs, electrocardiogram, and chest radiograph would have been non-diagnostic.

The RUSH exam, like the extended focused assessment with sonography for trauma, is used to identify pathology that requires immediate intervention.^1^–^3^ Because time is of the essence, each component of the RUSH exam is designed to answer a specific clinical question. This includes evaluation for reduced ejection fraction, signs of right heart strain, the state of the IVC and aorta, and the presence of pericardial effusion, free intraperitoneal fluid, pneumothorax, pleural effusion/hemothorax, and pulmonary edema.^4^ Despite the significant impact of point-of-care ultrasound on patient care, physicians who are further out from training have at times been reluctant to adopt its use; hence, education and training are still needed.^5^ Our case demonstrates how point-of-care ultrasound can rapidly identify a life-threatening pathology.

**CPC-EM Capsule**

What do we already know about this clinical entity?

*Point-of-care rapid ultrasound for shock and hypotension (RUSH) can identify dangerous pathology at the bedside.*

What is the major impact of the image(s)?

*This image shows the importance of RUSH in the undifferentiated hypotensive patient and the ability to rapidly diagnose cardiac tamponade.*

How might this improve emergency medicine practice?

*Routine use of RUSH can allow for faster diagnosis in critically ill patients.*

## Supplementary Information

Video Legend Point-of-care rapid ultrasound for shock and hypotension exam videos showing parasternal long, parasternal short, apical four chamber, subxiphoid and inferior vena cava views, respectively.
*RV*, right ventricle: *LV*, left ventricle: *LA*, left atrium; *RA*, right atrium; *IVC*, inferior vena cava.
